# Predictive Potential of Contrast-Enhanced MRI-Based Delta-Radiomics for Chemoradiation Responsiveness in Muscle-Invasive Bladder Cancer

**DOI:** 10.3390/diagnostics15070801

**Published:** 2025-03-21

**Authors:** Kohei Isemoto, Yuma Waseda, Motohiro Fujiwara, Koichiro Kimura, Daisuke Hirahara, Tatsunori Saho, Eichi Takaya, Yuki Arita, Thomas C. Kwee, Shohei Fukuda, Hajime Tanaka, Soichiro Yoshida, Yasuhisa Fujii

**Affiliations:** 1 Department of Urology, Institute of Science Tokyo, Tokyo 113-8519, Japan; 190051ms@tmd.ac.jp (K.I.); motohiro.fujiwara@gmail.com (M.F.); fukusho.uro@tmd.ac.jp (S.F.); hjtauro@tmd.ac.jp (H.T.); s-yoshida.uro@tmd.ac.jp (S.Y.); y-fujii.uro@tmd.ac.jp (Y.F.); 2 Department of Urology, Insured Medical Care Management, Tokyo Medical and Dental University, Tokyo 113-8519, Japan; 3 Department of Radiology, Institute of Science Tokyo, Tokyo 113-8519, Japan; kmrdrnm@tmd.ac.jp; 4 Department of Management Planning Division, Harada Academy, Kagoshima 891-0113, Japan; ffieldai@gmail.com; 5 Department of Radiological Technology, Kokura Memorial Hospital, Kitakyushu 802-8555, Japan; sahotatsu@gmail.com; 6 AI Lab, Tohoku University Hospital, Sendai 980-8574, Japan; takaya.wisdom0616@icloud.com; 7 Department of Radiology, Memorial Sloan Kettering Cancer Center, New York, NY 10065, USA; yukiarita1113@gmail.com; 8 Department of Radiology, Nuclear Medicine and Molecular Imaging, University Medical Center Groningen, Boston, MA 02114, USA; thomaskwee@gmail.com

**Keywords:** chemoradiotherapy, contrast media, machine learning, magnetic resonance imaging, urinary bladder neoplasms

## Abstract

**Background/Objectives**: Delta-radiomics involves analyzing feature variations at different acquisition time-points. This study aimed to assess the utility of delta-radiomics feature analysis applied to contrast-enhanced (CE) and non-contrast-enhanced (NE) T1-weighted images (WI) in predicting the therapeutic response to chemoradiotherapy (CRT) in patients diagnosed with muscle-invasive bladder cancer (MIBC). **Methods**: Forty-three patients with non-metastatic MIBC (cT2–4N0M0) who underwent partial or radical cystectomy after induction CRT were, retrospectively, reviewed. Pathological complete response (pCR) to CRT was defined as the absence of residual viable tumor cells in the cystectomy specimen. Identical volumes of interest corresponding to the index bladder cancer lesions on CE- and NE-T1WI on pre-therapeutic 1.5-T MRI were collaboratively delineated by one radiologist and one urologist. Texture analysis was performed using “LIFEx” software. The subtraction of radiological features between CE- and NE-T1WI yielded 112 delta-radiomics features, which were utilized in multiple machine-learning algorithms to construct optimal predictive models for CRT responsiveness. Additionally, the predictive performance of the radiomics model constructed using CE-T1WI alone was assessed. **Results**: Twenty-one patients (49%) achieved pCR. The best-performing delta-radiomics model, employing the “Extreme Gradient Boosting” algorithm, yielded an area under the receiver operating characteristic curve (AUC) of 0.85 (95% confidence interval [CI]: 0.75–0.95), utilizing four signal intensity-based delta-radiomics features. This outperformed the best model derived from CE-T1WI alone (AUC: 0.63, 95% CI: 0.50–0.75), which incorporated two morphological features and one signal intensity-based radiomics feature. **Conclusions**: Delta-radiomics analysis applied to pre-therapeutic CE- and NE-MRI demonstrated promising predictive ability for CRT responsiveness prior to treatment initiation.

## 1. Introduction

Recent guidelines now advocate for a multimodal bladder-sparing approach as an alternative for patients with muscle-invasive bladder cancer (MIBC) [1,2]. This alternative treatment typically consists of initial transurethral resection (TUR) followed by chemoradiotherapy (CRT), with the responsiveness to CRT serving as a pivotal determinant of oncological outcomes [3,4]. Therefore, the accurate prediction of CRT responsiveness prior to treatment initiation is imperative for identifying suitable candidates for bladder-sparing therapy.

In contemporary clinical practice for bladder cancer, MRI has become increasingly indispensable for locoregional staging and post-treatment surveillance [5,6]. Technological advancements in MRI, such as improved resolution and contrast enhancement techniques [7], have significantly enhanced the accuracy of these assessments. Moreover, contrast enhancement patterns on dynamic contrast-enhanced (CE-) MRI reportedly reflect microvessel density of bladder cancer, with denser tumors exhibiting a higher risk of local recurrence risk after TUR [8,9]. Radiomics, which analyzes tumor characteristics using radiological images, has gained prominence in the diagnosis and treatment of various cancers [10,11]. Within this field, delta-radiomics, which focuses on temporal variations in feature values, has emerged as a promising imaging biomarker [12,13]. Delta-radiomics features (ΔRFs) offer the potential for superior treatment assessment compared to single time-point features. This was demonstrated in locally advanced rectal cancer, wherein delta-radiomics analysis on presurgical MRI images acquired before and after neoadjuvant CRT predicts pathological complete response (pCR) [14]. Importantly, delta-radiomics was shown to enhance reproducibility and robustness by neutralizing variations in quantitative imaging features through subtraction, provided that acquisition parameters remain consistent across time points [15]. This inherent robustness makes delta-radiomics particularly suitable for longitudinal imaging studies, where variability in acquisition protocols can otherwise compromise feature reliability. Predicting treatment responsiveness prior to initiation is crucial in determining MIBC treatment strategies [16]. Delta-radiomics allows for the evaluation of temporal changes through subtraction analysis. Therefore, we hypothesized that analyzing pre- and post-contrast pretherapeutic MRI images utilizing delta-radiomics could enhance the assessment of tumor spatial heterogeneity.

In this study, we used a cohort that underwent partial or radical cystectomy following CRT, conducted based on a prospectively set treatment protocol to develop a model for predicting CRT responsiveness in patients with MIBC. We employed delta-radiomics adapted to contrast-enhanced and non-contrast-enhanced (NE-) fat-suppressed T1-weighted images (WI). In widely practiced bladder-preserving therapy using CRT, partial or radical cystectomy is rarely performed after CRT. Therefore, the analysis using this cohort, which uniquely allows for histopathological evaluation of CRT treatment effects, is considered highly valuable. By incorporating histopathological findings as a high-quality reference standard, this study offers robust insights into the predictive performance of delta-radiomics. Furthermore, we compared the predictive ability of the delta-radiomics model with that of a radiomics model constructed using only contrast-enhanced T1WI.

## 2. Materials and Methods

### 2.1. Patients

**Figure 1:**
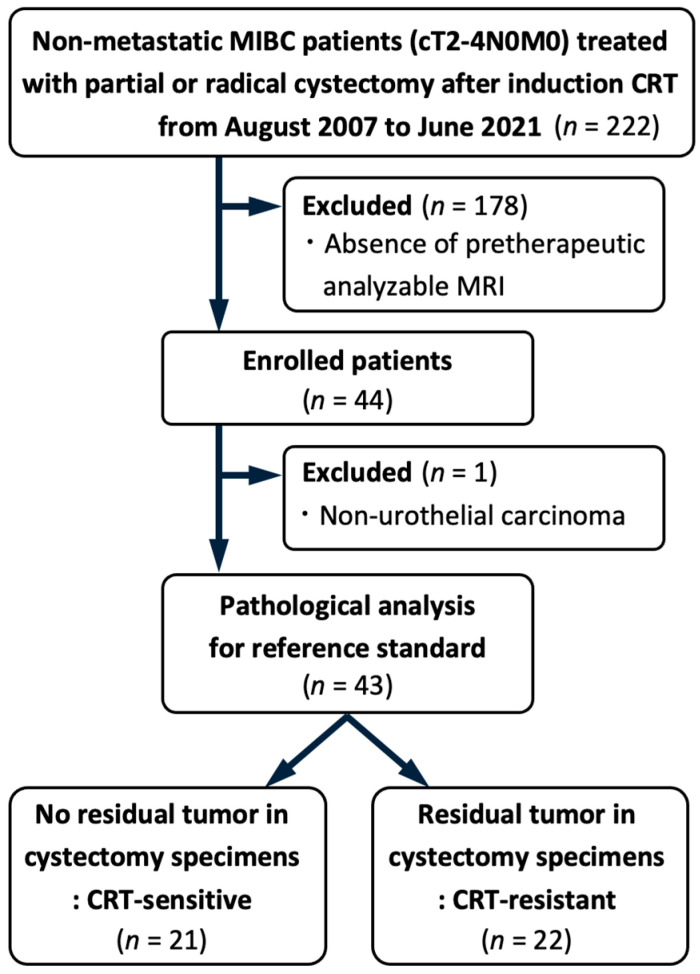
Study participant flowchart. MIBC, muscle-invasive bladder cancer; CRT, chemoradiotherapy; MRI, magnetic resonance imaging.

The Institutional Review Board at our center approved this study (M2021-223), and the need for written informed consent was waived owing to the retrospective nature of the study. Of the 222 consecutive patients who underwent partial or radical cystectomy after induction CRT for non-metastatic MIBC (cT2-4N0M0) between August 2007 and June 2021, we selected 44 patients who had undergone MRI examinations using the same protocol before initial TUR. Furthermore, we excluded one patient with non-urothelial carcinoma, resulting in a final analysis cohort of 43 patients. Figure 1 illustrates the patient selection process. The participants were treated based on a previously described therapeutic protocol [17,18]. In summary, patients underwent maximal TUR, defined as the complete endoscopic resection of all visible bladder tumors with no evidence of residual endophytic disease as confirmed cystoscopically, followed by CRT, which included external beam radiotherapy to the small pelvis (40 Gy) and concurrent intravenous cisplatin (20 mg/day for 5 days, administered in two cycles with a 3-week interval). After CRT, considering the initial tumor’s extent and localization within the bladder and the post-CRT status, patients were recommended to receive partial or radical cystectomy with standard pelvic lymph-node dissection for curative intent.

### 2.2. Data Collection

Clinical data for the patients, encompassing age, sex, clinical T stage, type of cystectomy, and TUR findings such as index tumor size and multiplicity, were extracted from electronic medical records.

### 2.3. Pathological Analysis and CRT Response Evaluation

The entire surgical specimens obtained from partial or radical cystectomy were fixed in 10% neutral buffered formalin and sectioned at 5 mm intervals. Each section was further cut into thin slices of 3 μm for histopathological evaluation. The histological grading of the specimens adhered to the 1973 World Health Organization classification [19], whereas the pathological T and N stages were assigned based on the 2002 TNM classification system [20]. pCR was defined as the complete absence of viable tumor cells in the specimen obtained from the cystectomy. Tumors achieving pCR after CRT were categorized as CRT-sensitive, whereas those not achieving pCR were considered CRT-resistant.

### 2.4. MRI Protocol

MRI examinations were conducted using a 1.5-T MRI system (Intera Achieva; Philips, Amsterdam, The Netherlands) equipped with a four-channel sensitivity-encoding body coil without breath-holding sequences. The standard MRI protocol comprised routine T1WI, T2WI, and diffusion-WI. The MRI protocol is summarized in Table 1. Dynamic CE-MRI was performed using an axial or sagittal fat-suppressed three-dimensional volumetric spoiled gradient-echo sequence (THRIVE during the first period [2007–2016]/e-THRIVE during the second period [2017–2021]) before and after the intravenous injection of gadolinium-based contrast agents. Gadoterate meglumine (Magnescope; Guerbet, Villepinte, France) was administered during the first period and gadobutrol (Gadovist; Bayer Schering Pharma, Leverkusen, Germany) during the second period. The dose and flow rate for each gadolinium-based contrast agent were 0.2 mL/kg, 3.0 mL/s and 0.1 mL/kg, 1.5 mL/s, respectively. The timing of image acquisition after injection was 45, 90, and 120 s for each period. Among these, the 90 s images, representing the venous phase, were selected for CE-T1WI analysis as this phase is optimal for evaluating tumor characteristics such as vascularity and tissue composition [5,21].

### 2.5. Delta-Radiomics Analysis

**Figure 2:**
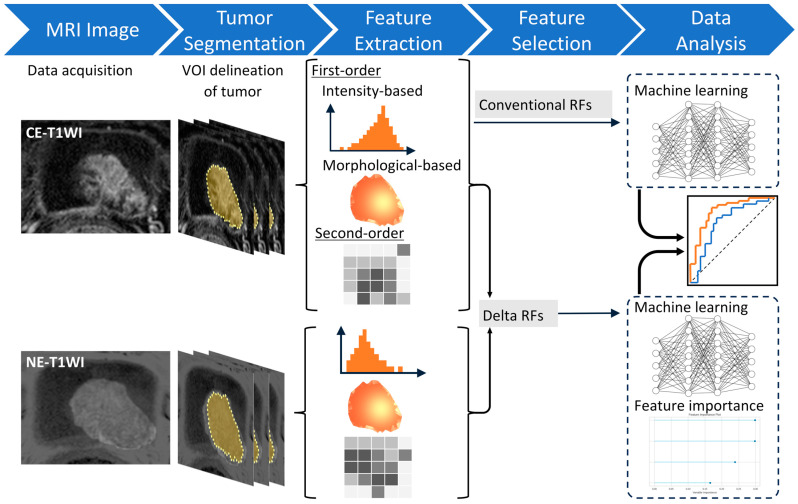
The workflow of necessary steps in the current study. CE, contrast-enhanced; NE, non-enhanced; WI, weighted image; VOI, volume of interest; RF, radiomics feature.

First, univariable logistic regression analysis was performed to evaluate the association between NE-T1WI, CE-T1WI, and ΔRFs with treatment response. A flow diagram of the delta-radiomics analysis is shown in Figure 2. Radiomics feature (RF) extraction and tumor segmentation of MIBC were performed using texture analysis software (LIFEx version 5.10; Inserm, Orsay, France) [22]. A urologist (Y.W.) initially delineated the regions of interest encompassing the visible tumor on axial CE-T1WI slice-by-slice basis to determine the volume of interest (VOI) for the primary tumor. An experienced radiologist (K.K.) subsequently reviewed and confirmed the VOI. Both Y.W. and K.K. were informed of the MIBC diagnosis but remained blinded to the patients’ CRT response. The finalized VOI was then applied to NE-T1WI. In cases of multiple tumors, the index tumor with the highest clinical T stage or, if equal, the largest lesion was selected. A total of 112 RFs were extracted from each VOI, including 23 first-order intensity-based indices, 14 first-order three-dimensional morphological indices, and 75 second-order features derived from the gray-level co-occurrence matrix, neighborhood gray-level different matrix, gray-level zone length matrix, and gray-level run-length matrix.

Subsequently, ΔRFs were calculated using the formula: ΔRFs = (RFCE − RFNE)/RFNE, where RFCE and RFNE represent the RFs extracted from CE- and NE-T1WI, respectively. Furthermore, a machine-learning prediction model was developed to predict pCR after CRT, utilizing the ΔRFs as explanatory variables. The predictive performance of this delta-radiomics model was compared with that of a radiomics model based on CE-T1WI alone.

### 2.6. Model Development and Statistical Analyses

In the machine-learning analysis, candidate explanatory variables were selected based on their correlation and multicollinearity. Fifteen representative machine-learning models were compared: Naive Bayes, Random Forest classifier, Linear Discriminant Analysis, Ridge classifier, K-Neighbor classifier, Extra Trees classifier, AdaBoost classifier, logistic regression, Light Gradient-Boosting Machine, Quadratic Discriminant Analysis, gradient boosting classifier, Extreme Gradient Boosting (XGBoost version 1.7.6), Decision Tree classifier, support vector machine with a linear kernel, and dummy classifier. These models were selected based on their high accuracy in binary classification tasks and their prevalence in the previous literature, and all were used with default parameter settings to avoid overfitting. The maximum tree depth was fixed at 7 to control model complexity, while the number of estimators was set to 140 to ensure sufficient boosting iterations. To prevent overfitting, a feature subsampling ratio of 0.5 and a data subsampling ratio of 0.9 were applied during training.

Ten-fold cross-validation was employed to evaluate model performance, and discrimination was assessed using the mean values of the area under the receiver operating characteristic curve (AUC). The accuracy, recall, precision, and F1 scores were also calculated from the evaluation metrics. Additionally, the feature importance of each selected ΔRF on the optimal prediction model was quantified. Categorical data are presented as numbers (percentages) and were compared using Fisher’s exact test, while continuous variables are reported as medians (interquartile range [IQR]) and were compared using the Wilcoxon rank-sum test. Statistical analyses were performed using Python (version 3.10.12; Python Software Foundation, Wilmington, DE, USA) and R (version 4.0.3; R Foundation for Statistical Computing, Vienna, Austria). Statistical significance was set at *p* < 0.05.

## 3. Results

### 3.1. Patient and Tumor Characteristics

The baseline characteristics of the eligible patients are listed in Table 2. The median patient age was 68 years (IQR 63–73 years). The cohort comprised of 13 patients (30%) with cT2 disease, 28 (65%) with cT3 disease, and 2 (5%) with cT4 disease. Fifteen patients (35%) had multiple tumors with different stages, and one (2%) had concomitant carcinoma in situ. After induction CRT, 25 (58%) and 18 (42%) patients underwent partial and radical cystectomies, respectively. Twenty-one patients (49%) achieved pCR and were classified as CRT-sensitive. The remaining 22 (51%) were classified as CRT-resistant, of whom 1 (2%) demonstrated pathological N1 disease.

### 3.2. Clinicopathological Variables Associated with CRT Sensitivity

There were no significant differences between CRT-sensitive and CRT-resistant MIBCs in clinicopathological parameters including age, sex, clinical T stage, index tumor size, multiplicity, presence of concomitant carcinoma in situ, and tumor grade (Table 1).

### 3.3. Diagnostic Performance for CRT Sensitivity of Models

**Figure 3:**
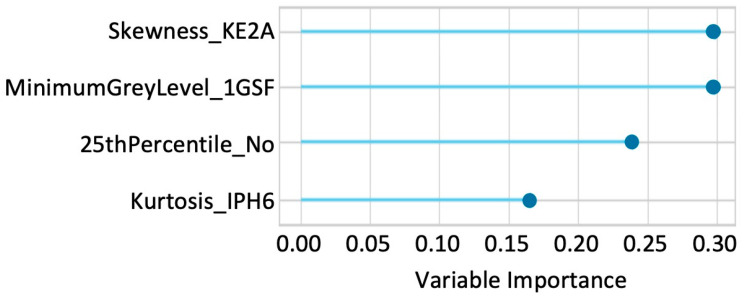
Illustration of the feature importance plot of the top four most influential variables in the best machine learning algorithm (XGBoost) for predicting treatment response. The average impact on model output magnitude is displayed, with higher values indicating greater feature importance.

Univariable logistic regression analysis was conducted to assess the association of NE-T1WI, CE-T1WI, and ΔRFs with treatment response, but no significant variables were observed. In the machine-learning analysis of the delta-radiomics model, considering correlation and multicollinearity, the candidate explanatory variables were 23 intensity-based ΔRFs. The optimal model, utilizing XGBoost, yielded an AUC of 0.85 (95% confidence interval [CI]: 0.75–0.95; recall: 0.80; accuracy: 0.57; precision: 0.58; F1 score; 0.63). This model incorporated four signal intensity-based ΔRFs: “Skewness”, reflecting a departure from normality; “MinimumGreyLevel”, indicating minimum intensity in the VOI; “25thPercentile”, representing the 1st quartile of signal intensity; and “Kurtosis”, reflecting the sharpness of the histogram of signal intensity. The feature importance of the selected variables in the XGBoost algorithm is illustrated in Figure 3.

For the radiomics model derived from CE-T1WI alone, the highest AUC was achieved through the machine-learning analysis utilizing XGBoost (AUC: 0.63; 95% CI: 0.50–0.75; recall: 1.00; accuracy: 0.60; precision: 0.60; F1 score: 0.74), incorporating two morphological features and one signal intensity-based feature: “volume”, “asphericity”, and “interquartile range” (denoting the difference in signal intensity between the upper and lower quartiles).

## 4. Discussion

The utilization of delta-radiomics, involving the subtraction of CE- and NE-T1WI features, enhanced the predictive performance for CRT responsiveness compared to conventional radiomics analysis based on CE-T1WI alone. Although the precision of 0.58 for the delta-radiomics model was comparable to that of 0.60 for conventional radiomics, it indicates room for clinical improvement. Nevertheless, the recall value of the delta-radiomics model was good at 0.80, which is clinically significant for selecting patients expected to respond to CRT while reducing false negatives. This study highlighted the potential of delta-radiomics analysis adapted to pre- and post-contrast T1WI for predicting the therapeutic response to CRT in MIBC prior to treatment initiation. The approach employed here was deemed to accurately reflect the vascular distribution within bladder tumors, indicating a correlation between vascular structure and CRT response.

Angiogenesis plays a pivotal role in tumor progression and metastasis, as it facilitates the supply of nutrients and oxygen to rapidly growing cancer cells. Immunohistochemical studies have consistently demonstrated that microvessel density is significantly correlated with various clinical parameters in bladder cancers, including recurrence and mortality rates [23,24]. This correlation underscores the importance of angiogenesis as a prognostic marker in bladder cancer management. Dynamic CE-MRI provides a non-invasive method to assess increased vascularity in tumors, heightened permeability of tumor capillaries, and an expansive extracellular compartment within the tumor tissue [25,26]. The contrast enhancement pattern serves as an indicator of microvessel density and has proven to be a valuable tool for predicting prognosis through the radiological assessment of tumor angiogenesis in bladder cancer [8]. A previous study showed that the contrast enhancement pattern of pre-therapeutic dynamic CE-MRI can effectively predict the presence of persistent or recurrent bladder cancers up to 1 year after radiation therapy following TUR [27]. In this study, delta-radiomics analysis utilizing pre-therapeutic CE- and NE-T1WI successfully integrated vascular information captured by dynamic CE-MRI into a quantitative framework. By enabling a more precise assessment of tumor properties from pre-intervention images, delta-radiomics facilitates the evaluation of vascular distribution within bladder tumors. This approach is particularly critical for tailoring treatment strategies, as vascular characteristics of tumors are associated with their response to CRT.

RFs extracted from single-time-point images provide valuable insights into tumor characteristics. However, post-treatment changes such as inflammation and fibrosis can result in irregular bladder wall thickening, producing high signal intensity on T2WI [5]. This poses a challenge in differentiating bladder cancer from benign treatment-related changes, leading to low sensitivity, specificity, and accuracy of T2WI in predicting pCR following CRT. Consequently, the predictive performance of multiparametric MRI for therapeutic response assessment after CRT was reported to be suboptimal for T2WI and dynamic CE-MRI, whereas diffusion-WI has demonstrated superior sensitivity and accuracy [28]. To address this limitation, the transformation of RFs across multiple time-points, termed delta-radiomics, has emerged as a promising approach [29]. Delta-radiomics enables the quantification of temporal tumor alterations, offering enhanced prognostic utility compared to single-time-point RFs. Despite its potential, radiomics analysis, particularly texture analysis, faces significant challenges related to reproducibility and robustness [30,31]. Variability in imaging acquisition parameters is a major source of uncertainty in MRI RFs. This variability arises from three primary factors: the absence of standardized intensity units in MRI, inconsistencies in acquisition protocols, and variations in reconstruction algorithms and settings. Unlike CT tomography, which represents image intensities in standardized physical units like Hounsfield units, MRI intensities are inherently non-standardized and influenced by tissue-specific properties. Additionally, variations in acquisition protocols and reconstruction algorithms can significantly alter image appearance and introduce artifacts. These factors collectively contribute to the variability and reduced reproducibility of RFs, particularly first-order and texture features. A recent study investigating the longitudinal acquisition repeatability of MRI RFs found that shape features exhibited the highest repeatability, while first-order and texture features were less consistent [32]. Delta-radiomics has demonstrated inherent robustness in a CT imaging study, as it neutralizes variations in quantitative imaging features through subtraction if the acquisition parameters are consistent across time points [15]. The use of identical protocols and reconstruction algorithms for pre- and post-contrast T1WI in this study ensured consistency in imaging acquisition, minimizing potential variability caused by differences in imaging protocols or reconstruction settings. This consistency enhanced the robustness of delta-radiomics by enabling a more precise neutralization of variations in quantitative imaging features. While previous studies have demonstrated the utility of single-time-point diffusion-weighted imaging in predicting treatment response in MIBC [33,34], our findings suggest that delta-radiomics analysis using pre- and post-contrast T1WI may address some limitations of single-time-point analyses and offer additional predictive advantages.

Previous studies employing delta-radiomics have primarily focused on assessing early post-treatment responses with the aim of optimizing sequential treatment strategies [14,35,36]. This approach, while valuable for evaluating early treatment effects, relies on post-treatment imaging. However, in the context of bladder preservation therapy for MIBC, pre-treatment evaluation is crucial because radical surgery may not be an option owing to progression if CRT is found to be ineffective after treatment is initiated [16,37]. The neoadjuvant chemotherapy Vesical Imaging-Reporting and Data System (nacVI-RADS) is a recently proposed model that delineates response to neoadjuvant therapy using pre- and post-treatment MRI, and its clinical utility is highly anticipated [38]. The nacVI-RADS is defined through the integration of the pretreatment VI-RADS score with MRI findings obtained after neoadjuvant chemotherapy, which evaluate factors such as residual tumor presence, tumor size, and muscularis propria infiltration. It has been reported to be strongly associated with pathological downstaging and survival outcomes when applied after three courses of pembrolizumab as a neoadjuvant setting in patients with MIBC [39]. The application of delta-radiomics for such early treatment response assessment is likely to be advantageous. However, within the clinical setting of MIBC management, evaluation prior to the commencement of neoadjuvant intervention is highly preferable. This study introduced an innovative approach by employing delta-radiomics for CE- and NE-T1WI prior to treatment intervention, which allows the precise assessment of spatial heterogeneity by mitigating confounding effects like inflammation through subtraction. Notably, no clinicopathological variables were found to be useful for predicting CRT responsiveness, highlighting the potential of delta-radiomics as a superior predictive tool. Despite the lack of RFs associated with tumor shape owing to the subtractive calculation method, delta-radiomics still outperformed conventional radiomics with CE-T1WI alone in predicting CRT responsiveness.

Our study has several potential limitations. This study focused solely on T1WI before and after contrast administration. The utility of apparent diffusion coefficient maps in predicting bladder tumor grade and response to CRT has been documented [33,34]. Further research is needed to identify appropriate MRI sequences and parameters. In addition, the limited sample size of 43 patients poses a challenge to the generalizability and conclusiveness of this model as a prognostic tool. Due to the small sample size, external validation of the model could not be performed, which limits the robustness of the findings. Nonetheless, the optimal predictive model in this study was constructed using four selected features, with the feature-to-sample ratio maintained below 1:10, a range generally considered appropriate for small sample sizes, thereby minimizing the risk of overfitting and enhancing the reliability of the internal validation [40]. However, it is important to note that cohorts undergoing partial or radical cystectomy after CRT based on prospective protocols are rare, as bladder-preserving therapy with CRT typically precludes the need for cystectomy. This rarity underscores the significance of our study, as it provides valuable insights into a unique patient group where the therapeutic effect of CRT could be evaluated histopathologically. Histopathological findings are considered a high-quality reference standard in the METhodological RadiomICs Score (METRICS) [41]. The METRICS score, a quality assessment tool established by international experts using a transparent methodology, aims to assess and enhance the quality of research in radiomics and machine learning. In this study, the METRICS score was rated as “good”. Therefore, we believe it is particularly significant that this study was conducted in a patient cohort where the therapeutic effects of CRT could be rigorously assessed through histopathological evaluation. Machine learning requires substantial amounts of data for training and testing, necessitating the accumulation and analysis of additional cases in future research. Further studies are required to validate the utility of delta-radiomics-based treatment effect predictions in large independent cohorts.

## 5. Conclusions

Delta-radiomics analysis employing pre-therapeutic CE- and NE-T1WI enabled the development of a predictive model for assessing the treatment response to CRT in patients with MIBC. Delta-radiomics, which exhibited superior predictive performance compared to conventional radiomics analyses relying on CE-T1WI alone, could be a promising imaging evaluation method for predicting treatment response and may potentially be useful for treatment optimization.

## Figures and Tables

**Table 1:** MRI scan protocols at each term.

	Achieva 1.5 T, *n* = 43					
	T2WI		DWI		DCE Imaging	
Period (year)	2007–2016	2017–2021	2007–2016	2017–2021	2007–2016	2017–2021
Type of sequence	2D-FSE	3D-FSE	SS-EPI	SS-EPI	3D-GRE	3D-GRE
Orientation	Axial	Axial	Axial	Axial	Axial	Axial or Sagittal
TR/TE (ms)	4500/100	1500/144	5000/80	5000/80	4.0–4.5/2.0–2.2	4.8/2.4
Flip angle (degree)	90	90	90	90	13	15
FOV (cm)	30	30	30	30	22–30	22
Matrix	512 × 512	512 × 512	256 × 256	256 × 256	288 × 288–512 × 512	288 × 288
Slice thickness (mm)	4.4–5.0	1.6	4.4–5.0	4.4	2.0–6.5	2.0
Slice gap (mm)	0.4–0.5	0	0.4–0.5	0.4	0	0
Number of excitations	2–3	1	2–6	3	1–2	1
*b*-value (s/mm^2^)	—	—	0, 500, 1000, 2000	0, 1000, 2000	—	—

T2WI, T2-weighted imaging; DWI, diffusion-weighted imaging; DCE, dynamic contrast-enhanced; 2D-FSE, two-dimensional fast spin-echo; 3D-FSE, three-dimensional fast spin-echo; SS-EPI, single-shot echo planar imaging; 3D-GRE, three-dimensional gradient-echo; TR/TE, repetition time/echo time; FOV, field of view.

**Table 2:** Patient and tumor characteristics of the 43 eligible patients according to CRT sensitivity.

Variables	*n* (%)	CRT-Sensitive (*n* = 21)	CRT-Resistant (*n* = 22)	*p* Value
Age, years *	68 (63–73)	69 (63–74)	67 (63–74)	0.60
Gender Male Female	32 (74) 11 (26)	18 (86) 3 (14)	14 (64) 8 (36)	0.10
Clinical T stage T2 T3 T4	13 (30) 28 (65) 2 (5)	2 (24) 3 (71) 4 (5)	8 (36) 13 (59) 1 (5)	0.67
Size of index tumor (cm) *	3.0 (2.0–4.0)	2.7 (2.0–5.1)	3.0 (2.0–4.0)	0.88
Multiplicity Yes No	15 (35) 28 (65)	5 (24) 16 (76)	10 (45) 12 (55)	0.14
Presence of concomitant CIS Yes No	1 (2) 42 (98)	1 (5) 20 (95)	0 (0) 22 (100)	0.49
Highest tumor grade Grade 2 Grade 3	1 (2) 42 (98)	1 (5) 21 (95)	0 (0) 22 (100)	0.49
Cystectomy after induction CRT Partial Radical	25 (58) 18 (42)	15 (71) 6 (29)	10 (46) 12 (55)	0.08
Pathologic T stage T0 Ta/is/1 T2 T3	21 (49) 9 (21) 3 (7) 10 (23)	21 (100) 0 (0) 0 (0) 0 (0)	0 (0) 9 (41) 3 (14) 10 (46)	<0.01
Pathologic N stage N0/x N+	42 (98) 1 (2)	21 (100) 0 (0)	21 (95) 1 (5)	0.51

* Median (interquartile range).

## Data Availability

The datasets presented in this article are not readily available because the data are part of an ongoing study. Requests to access the datasets should be directed to the corresponding author.
